# Underreporting of deaths in the maternal deaths surveillance system in one region of Morocco

**DOI:** 10.1371/journal.pone.0188070

**Published:** 2018-01-31

**Authors:** Saloua Abouchadi, Wei-Hong Zhang, Vincent De Brouwere

**Affiliations:** 1 Ecole Nationale de Santé Publique (ENSP), Rabat, Morocco; 2 School of Public Health, Université Libre de Bruxelles (ULB), Brussels, Belgium; 3 Maternal and Reproductive Health Unit, Department of public health, Institute of Tropical Medicine (ITM), Antwerp, Belgium; 4 Research Laboratory for Human Reproduction, Faculty of Medicine, Université Libre de Bruxelles (ULB), Brussels, Belgium; 5 WHO collaborating centre: International Centre for Reproductive Health (ICRH), Ghent University, Ghent, Belgium; Ege University, School of Medicine, TURKEY

## Abstract

**Objective:**

To assess the reliability of maternal deaths surveillance system (MDSS) and to determine the factors that influence its completeness in one region of Morocco.

**Methods:**

We conducted a retrospective survey in “Gharb Chrarda Bni Hssen” region (GCBH) between January the 1st, 2013 and September the 30th, 2014 using multiple sources approach. All deaths of women of reproductive age (WRA) were investigated using certificates with medical cause, medical records and interviews with household members and relatives to ascertain a pregnancy-related or maternal death. An External Expert Committee reviewed the information collected to assign a cause for each death. Our results were compared to those reported in the same period by the MDSS.

**Findings:**

Our study identified 690 deaths of WRA and 69 maternal deaths of which 34.8% occurred outside health facilities. The MDSS recorded during the study period 538 deaths of WRA and 29 maternal deaths (including only one outside health facility) representing respectively an underreporting of 22.0% and 58.0%. Late maternal deaths represented 11.4% of all deaths of women with a registered pregnancy within 12 months prior to the death, while the MDSS identified none. The maternal mortality ratio (MMR) was estimated at 103, approximately 2.5 times higher than that reported in the MDSS.

**Conclusion:**

Our study has shown weaknesses in the current notification system for maternal deaths in the region of GCBH. Therefore, more attention must be given to the regional committees in charge of auditing the cases and defining actions to be implemented to prevent further maternal deaths.

## Introduction

To achieve the Millennium Development Goals (MDGs), countries and international agencies were committed to monitor progress in maternal mortality reduction (MDG 5) [1, 2]. Thanks to this push, many countries made notable progress in data gathering through civil registration systems, surveys, censuses and specialized studies [1]. However, accurate measurement of maternal mortality remains challenging, particularly in developing countries [1–3].

During the transition to the Sustainable Development Goals, it has been proposed that progress towards ending preventable maternal deaths should continue to be measured by monitoring the maternal mortality ratio (MMR) [4–6]. Currently, there is consensus on the need to improve measurement, data quality and the importance of counting all maternal deaths [6]. The World Health Organization (WHO) together, with its associated agencies, launched the Maternal Death Surveillance and Response (MDSR) policy in 2012, as the new standard for a more accurate monitoring of countries’ progress [7–9]. The implementation of this system is now underway in 103 low and middle income countries [10]. However, the status of implementation varies widely between countries and many countries are experiencing significant challenges and barriers, such as inefficient and incomplete notification systems, the absence of a legal framework, a culture of blame, and lack of resources [10].

In Morocco, where the MMR decreased from 317 (80% uncertainty interval [UI] 241–387) in 1990 to 121 (80% UI 93–142) in 2015, i.e. a 62% reduction [1], the Ministry of Health implemented a nationwide maternal death surveillance system (MDSS) in June 2009 [11]. This system includes (i) a mandatory reporting system of all deaths of women of reproductive age (WRA) (15–49 years old); (ii) a preliminary survey to identify pregnancy-related deaths (PRDs); and (iii) a confidential enquiry into all PRDs in both communities and health facilities. The design and implementation process of MDSS have been detailed elsewhere [11, 12]. Two previous National Confidential Enquiries into Maternal Deaths in 2009 and 2010 estimated the MMR at 66 and 59, respectively [13, 14]. These estimates correspond to half of those announced by the Maternal Mortality Estimation Inter agency Group (MMEIG) [1]. Therefore, it is necessary to understand the difference between the estimates produced by the two systems: underestimation by MDSS or overestimation through the MMEIG statistical model? This study aimed to address the reliability of MDSS and to determine the factors that influence its completeness at regional level in Morocco.

## Methods

Morocco is a lower-middle income country in North Africa. In 2014, the population was about 33 million inhabitants with 60% living in urban areas and 27% being WRA [15]. The estimated annual number of deliveries was 664,000 and the proportion of institutional deliveries (including the private sector) was 72.7% at national level, with a wide disparity between regions (min-max 56.8% - 90.9%) [16, 17]. Until 2015, the country was divided into 16 administrative regions.

We performed a retrospective survey on women of reproductive age (WRA) in one region of Morocco between January the 1^st^, 2013 and September the 30^th^, 2014 using multiple sources approach in identifying PRDs and maternal deaths. The criteria used for selecting eligible regions for participation included: i) at least 30 000 expected births per year, to ensure inclusion of a sufficient number of maternal deaths; ii) a rural population of at least 50%, to ensure inclusion of home deaths; and iii) a proportion of institutional deliveries below the national average (less than 73%), to ensure inclusion of a sufficient number of home deliveries. Four regions met the eligibility criteria (S1 Table). We purposively selected “Gharb Chrarda Bni Hssen” (GCBH) region that is located in the Northwest of Morocco, covering an area of 8 800 km^2^, with three provinces (Kénitra, Sidi Kacem and Sidi Slimane) and 63 communes. In 2014, the total population was 1,904,112 inhabitants, with 52% of people living in rural areas [15]. The number of WRA resident in this region was around 500,000 and there were 37,000 annual expected live births [17, 18]. The proportion of institutional deliveries was 59% which corresponds to the second lowest proportion in the sixteen Moroccan regions [16]. According to the MDSS, GCBH had the lowest MMR (52 per 100,000 live births) among the eligible regions in 2010 [14].

Three stages were used for data collection to assess the completeness of maternal deaths reporting: 1) Identification of deaths among WRA, 2) Ascertainment of pregnancy related status and 3) Determination of causes of maternal deaths, including a qualitative data collection through field observations, documents reviews and informal discussions with health staff and managers, to investigate the process, strengths and challenges of implementing the MDSS.

Forty-one investigators were selected from health staff in primary health care facilities (nurses) based on their relationship with the local authorities and familiarity with the local community. A two-day training workshop was organized for familiarizing health workers with the details of data collection procedures, administering the verbal autopsy interview (attitude and questionnaire) and extraction of data using the forms.

### Identification of deaths of WRA

During the period between September and November 2014, we identified all deaths occurring among women aged 15–49 years who were resident and died in GCBH between January the 1^st^, 2013 and September the 30^th^, 2014. Women who were not resident in the study region were excluded. We used four existing data sources of death reporting in Morocco including 84 civil registration offices, 11 health offices and 14 health facilities (4 public hospitals and 13 private clinics), as well as local authorities (33 divisions called ‘Caidats’) (Table 1).

**Table 1:** Data sources in GCBH region.

Data source	Description	Data collected
Data source 1: Civil registration office	Available in each commune. Deaths have to be reported by the family to the local vital statistics officer within 30 days after the death. The death is registered in the civil registry only if the family presents a death certificate issued by the hospital, health office or local authority.	Full name, sex, nationality, occupation, marital status and address of the deceased, Date, hour and place of death, Date and place of birth, Names and address of parents, Name, age, occupation, address of the person who reported the death and its family relationship with the deceased.
Data source 2: Hospital register	When a woman dies in a hospital, a physician must fill in a death certificate including the cause of death.	Full name and nationality of the deceased Date, hour and place of death Date and place of birth, Names of parents, Date and department of admission in the hospital, **Cause of death**.
Data source 3: Health office	Communal technical service that records deaths occurring in its serviced area (hospitals, home, and other places). The health office validates the hospital death certificate before the family is allowed to bury the deceased. For deaths occurring outside the hospital, the health office produces the death certificate. In addition, the health office investigates deaths that are suspected to be violent, unnatural or of unknown causes.	Full name, sex, age and nationality of the deceased, Date, hour and place of death, **Cause of death**.
Data source 4: Local authority	In some rural areas where there is no health office, the death certificate is issued by the local authority that represents the Ministry of Interior, in charge of local administration	Full name and address of the deceased, Date and place of death, Date and place of birth, Name and address of the person who reported the death.

A search for duplicates was performed manually by classifying the file data sequentially in chronological order according to the name of women followed by date and place of death. The deaths were considered duplicates when these variables were strictly identical. In case of doubt, the principal investigator (SA) verified the name of the parents of the deceased. When some data was missing but the date and place of death were the same, the death record was reviewed to make sure it was not a duplicate.

### Ascertainment of maternal/pregnancy related status

We conducted interviews between February and August 2015 with respondents from the household of the deceased WRA. The investigator began with a preliminary survey to identify deaths occurring during pregnancy or within one year of pregnancy regardless of the cause of death or relation between pregnancy and the death [19]. Then, a verbal autopsy was carried out using the WHO verbal autopsy questionnaire, adapted to the Moroccan context and tested prior to use [20]. The verbal autopsy interviewers had no clinical information on the death or on the cause of death.

Between December 2015 and February 2016, an investigation of the public hospitals’ medical records was performed by the principal investigator (SA) to collect further data on the medical causes of the deaths among WRA identified through the hospital registers.

An External Expert Committee consisting of three medical doctors (an obstetrician gynecologist, an anesthetist & a public health specialist) was set up to review the information collected from the hospitals, health offices and families and assign a medical cause for each death (pregnancy related, maternal or not). Pregnancy-related deaths, maternal deaths and late maternal deaths were defined according to the International Classification of Diseases, tenth revision [19]. The results of our study were compared to those reported in the same period by the MDSS in the study region. Statistical analysis was performed using Epi info 7.

The MMR was calculated by dividing the number of maternal deaths occurring in a period by the number of live births occurring in the same period [1]. As accurate data on live births from the civil registration system were not available, we used estimates provided by the Ministry of Health [17, 18]. The number of live births estimated in GCBH during the 21-month period was 66,687.

### Ethical approval

This study was approved by the Institutional Review Board of the Institute of Tropical Medicine of Antwerp (No. 957/14), the Ethics Committee for Biomedical Research of the Faculty of Medicine and Pharmacy of Rabat (No. 702/14) and the National Control Commission for the Protection of Personal Data in Morocco (No. A-R-159/2014).

## Results

### Deaths among WRA

During the 21-month period, we identified 1357 WRA’s deaths by combining the four data sources. After record linkage, 549 records were identified as duplicates and removed. Among the remaining 808 deaths, we excluded a further 24 duplicates based on interview with families, as well as 94 deaths that did not meet the inclusion criteria (34 cases occurred out of the study period, 21 deaths had unknown age or age not between 15–49 years and 39 women were resident outside the study region). Finally, 690 deaths of WRA were included in the analysis.

**Fig 1:**
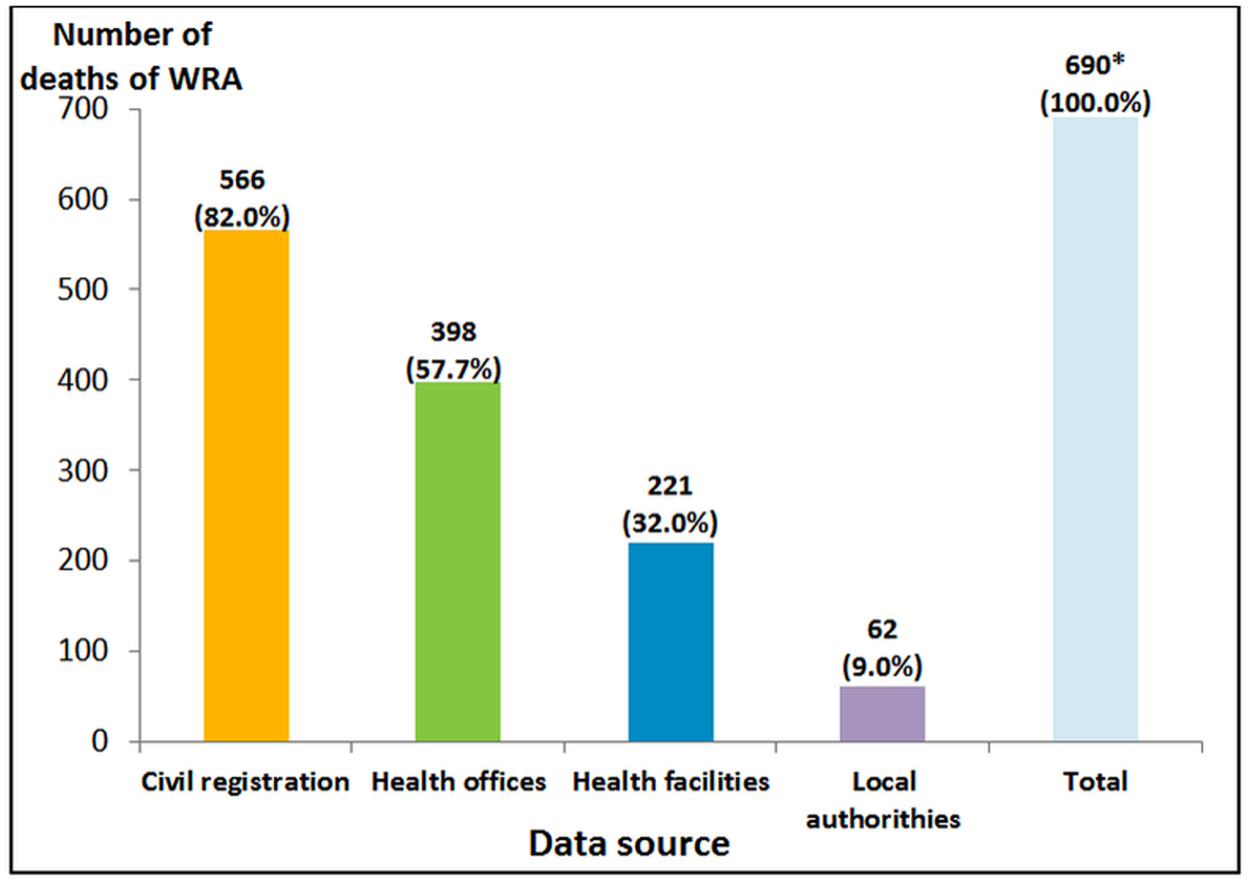
Deaths among WRA identified by data source (N = 690). *One death not recorded by data source in GCBH but identified through verbal autopsies in Sidi Kacem. It was a woman resident in GCBH but who died outside of the region thus not recorded in any of the four data sources mentioned.

Overall, civil registration was the most comprehensive source as it accounted for 82.0% of deaths, in contrast with local authorities who reported only 9.0% of deaths (Fig 1).

**Fig 2:**
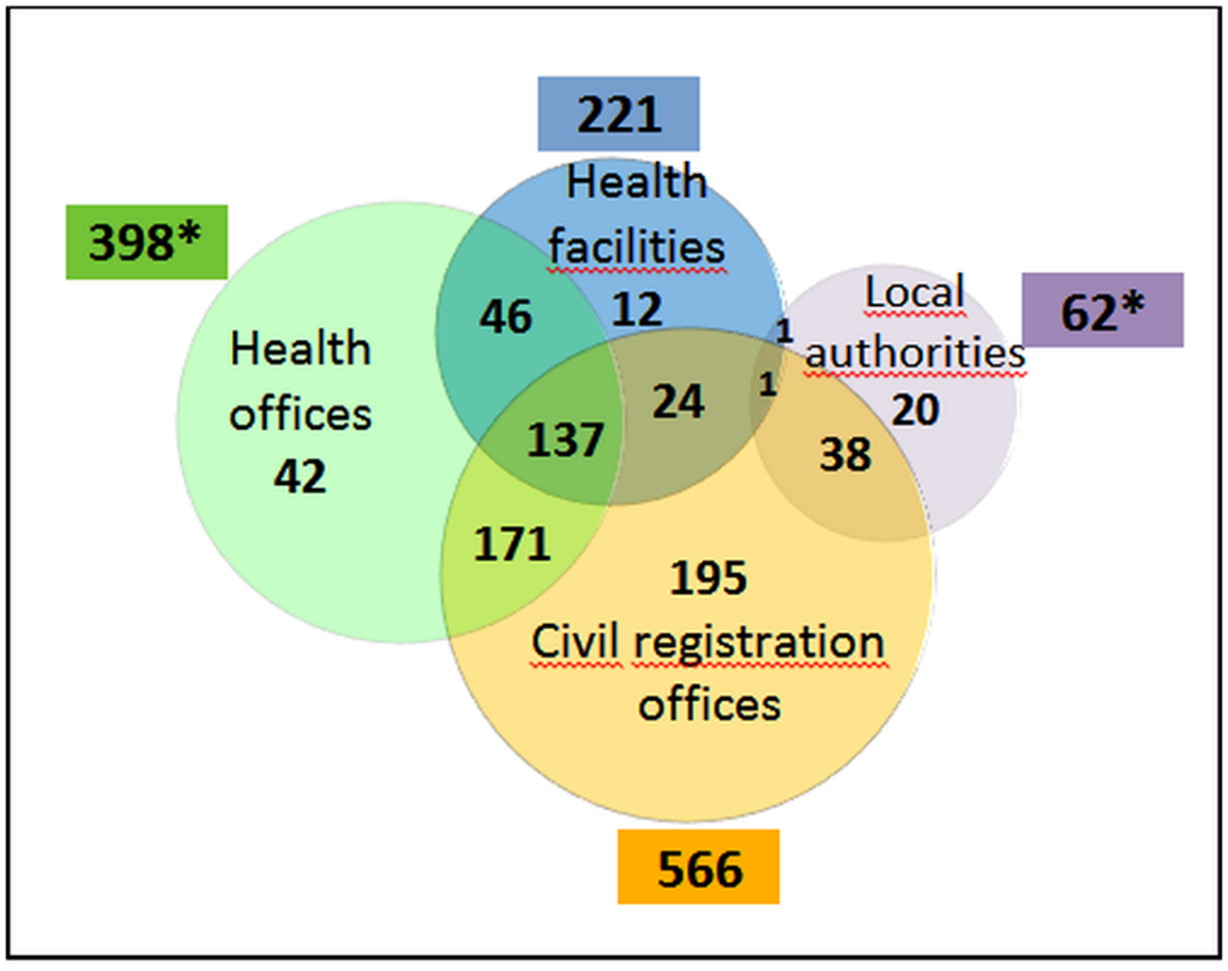
Deaths among WRA after record linkage between data sources (N = 690). *including 2 other deaths identified by both health offices and local authorities.

Twenty per cent of deaths were identified by three sources, 40.9% by two sources and 39.0% by one source (Fig 2).

Regarding the place of death, 436 deaths (63.2%) occurred outside health facilities and 205 (29.7%) in a health facility (including 5 deaths during transfer between health facilities). For 49 cases (0.7%), the place of death was not known (S2 Table). We identified 4 deaths that occurred outside the region.

**Fig 3:**
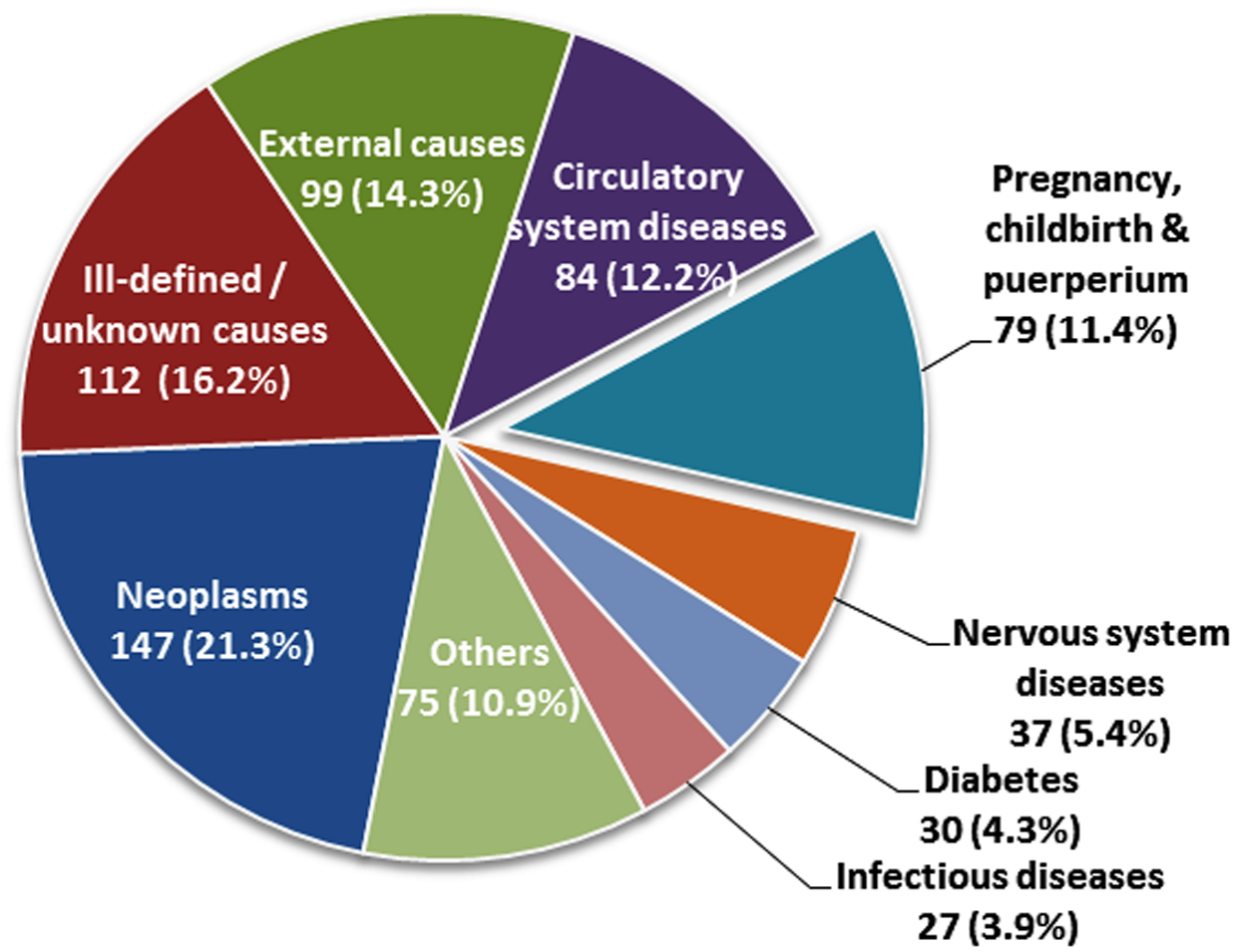
Causes of deaths among WRA (N = 690).

The leading cause of death of WRA was cancer (21.3%) (Fig 3). The most common types were breast (36.1%) and cervical (14.3%) cancer. Cardiovascular and cerebrovascular diseases represented 12.2% of all deaths of WRA. External causes (including road accident, suicide, assault, complications of medical care and poisoning) accounted for 14.3%. However, 112 deaths (16.2%) were classified as ill-defined or as unknown cause due to incomplete information (Fig 3).

### Deaths during pregnancy or within one year of pregnancy

Among the 690 deaths of WRA, a cause of death was only mentioned in the hospitals’ and health offices’ registers (436 cases). We identified through the review of these registers 34 PRDs; the other 402 deaths were classified as non-pregnancy-related. In parallel, interviews with the families of 515 of the 690 identified deaths of WRA were carried out. Through these verbal autopsies, we further identified 31 additional PRDs and 9 late maternal deaths. Among the 175 families not interviewed, the address was not mentioned or incomplete for 80 women and false in 51 cases. In 10 cases, we did not find anyone at the address indicated. Twenty four families had moved (8 of them outside of the region) and the new address was not found. Ten families refused to participate in our study. We also reviewed 205 hospital medical records and registers of hospital departments to confirm and complete the cause of WRA deaths that occurred in the health facilities and identified 5 additional PRDs.

Overall, there were 690 deaths of WRA identified, of which 79 (11.4%) occurred during pregnancy or within one year of pregnancy, 401 (58.1%) were non-pregnancy-related, and for 210 (30.4%) the pregnancy status was not clearly stated. Among those, 106 deaths occurred at home or during transfer from home to a health facility, 65 in health facilities and for 39 cases, the place of death was not known.

Forty-seven percent of deaths occurred during childbirth and within the first 24 hours postpartum. Another quarter (25.3%) occurred during the postpartum/postabortum period, after 24 hours but prior to 42 days, and 11.4% occurred between 43 days and one year after the pregnancy ended. There were 11 PRDs (13.9%) that occurred in the antepartum period (Table 2).

**Table 2:** Time and place of deaths (N = 79).

Period of death	Place of death		TOTAL N (%)
	Health facility (including transfer) n (%)	At home, other n (%)	
**During pregnancy**	**3 (5.9)**	**5 (17.9)**	**8 (10.1)**
> 22 weeks	2 (3.9)	5 **(17.9)**	7 (8.9)
Unknown*	1 (2.0)	0 (0.0)	1 (1.3)
**During delivery** (>22 weeks)	**2 (3.9)**	**1 (3.6)**	**3 (3.8)**
**Postpartum**	**40 (78.4)**	**19 (67.9)**	**59 (74.7)**
≤ 24 h	27 (52.9)	7 (25.0)	34 (43.0)
> 1 d and ≤ 42 d	7 (13.7)	9 (32.1)	16 (20.3)
Unknown (but ≤ 42 d) **	2 (3.9)	0 (0.0)	2 (2.5)
> 42 d	4 (7.8)	3 (10.7)	7 (8.9)
**Postabortum**	**5 (9.8)**	**1 (3.6)**	**6 (7.6)**
> 1 d and ≤ 42 d	4 (7.8)	0 (0.0)	4 (5.1)
> 42 d	1 (2.0)	1 (3.6)	2 (2.5)
Unknown**	**1 (2.0)**	**2 (7.1)**	**3 (3.8)**
**Total**	**51 (100.0)**	**28 (100.0)**	**79 (100.0)**

*Death due to road accident; the husband didn’t know the age of the pregnancy.

**Incomplete information in medical record and/or verbal autopsy not done because family not found.

The majority of deaths during pregnancy or within one year of termination of pregnancy (49 i.e. 62.0%) occurred in public health facilities (including 3 deaths during transfer and one death in a basic health service). Two deaths took place in a private clinic and 28 deaths (35.4%) occurred outside health facilities.

### Maternal deaths

Sixty-nine PRDs were classified as maternal deaths. At the time of death, the average age of women was 31 years. The majority of women (97.0%) were married, 87.1% (54) were engaged in unpaid work at home and 60.3% (41) lived in a rural area.

Among the 52 women who died after childbirth, 32 (61.5%) had a vaginal delivery, while 15 (28.8%) had a caesarean section. For 5 women (9.6%), no information was available. Of the 50 women for whom the place of childbirth was specified, 62.0% gave birth in a public hospital, 30.0% at home and 4.0% either in private clinics or basic health services.

The majority of maternal deaths (84.1%) were due to direct causes, 10.1% to indirect causes and for 5.8% the cause was unknown. The most common diagnoses were hemorrhage (39.1%) and hypertensive disorders (23.2%). We identified 4 deaths (5.8%) due to abortion, all of which occurred in public hospitals. Deaths due to direct causes occurred mostly (72.4%) in health facilities, whereas those due to indirect causes occurred mainly at home (Table 3).

**Table 3:** Number of maternal deaths and late maternal deaths by site of death and according to group of causes.

Cause of death	Place of death		Total N (%)
	Health facility (including transfer) n (%)	At home, other n (%)	
**Maternal deaths**	**45 (65.2)**	24 **(34.8)**	**69 (100.0)**
• Direct cause	42 (72.4)	16 (27.6)	58 (84.1)
Hemorrhage	21 (77.8)	6 (22.2)	27 (39.1)
Hypertensive disorder	10 (62.5)	6 (37.5)	16 (23.2)
Abortion	4 (100.0)	0 (0.0)	4 (5.8)
Infection	2 (66.7)	1 (33.3)	3 (4.3)
Venous thromboembolism	1 (33.3)	2 (66.7)	3 (4.3)
Complications of medical/surgical care	2 (100.0)	0 (0.0)	2 (2.9)
Unspecified direct cause	2 (66.7)	1 (33.3)	3 (4.3)
• Indirect cause	2 (28.6)	5 (71.4)	7 (10.1)
Cardiopathy	0 (0.0)	2 (100.0)	2 (2.9)
Cancer	0 (0.0)	1 (100.0)	1 (1.4)
Other	2 (50.0)	2 (50.0)	4 (5.8)
• Unknown/ Undetermined	1 (25.0)	3 (75.0)	4 (5.8)
**Others**	**6 (60.0)**	**4 (40.0)**	**10 (100.0)**
• Incidental cause	1 (100.0)	0 (0.0)	1 (10.0)
• Late maternal deaths	5 (55.6)	4 (44.4)	9 (90.0)
**TOTAL**	**51 (64.6)**	**28 (35.4)**	**79 (100.0)**

We identified one death due to an incidental cause (road accident) and 9 late maternal deaths, of which 5 occurred between 43 days and 3 months after delivery. Two deaths were due to direct causes (post-abortion cerebral thrombosis and peritonitis post C-section), 5 were due to indirect causes (cardiopathy, malignant tumor of the cervix, stomach cancer, hemorrhagic stroke) and the cause was undetermined for 2 cases.

### Comparison between study results and data from MDSS

**Fig 4:**
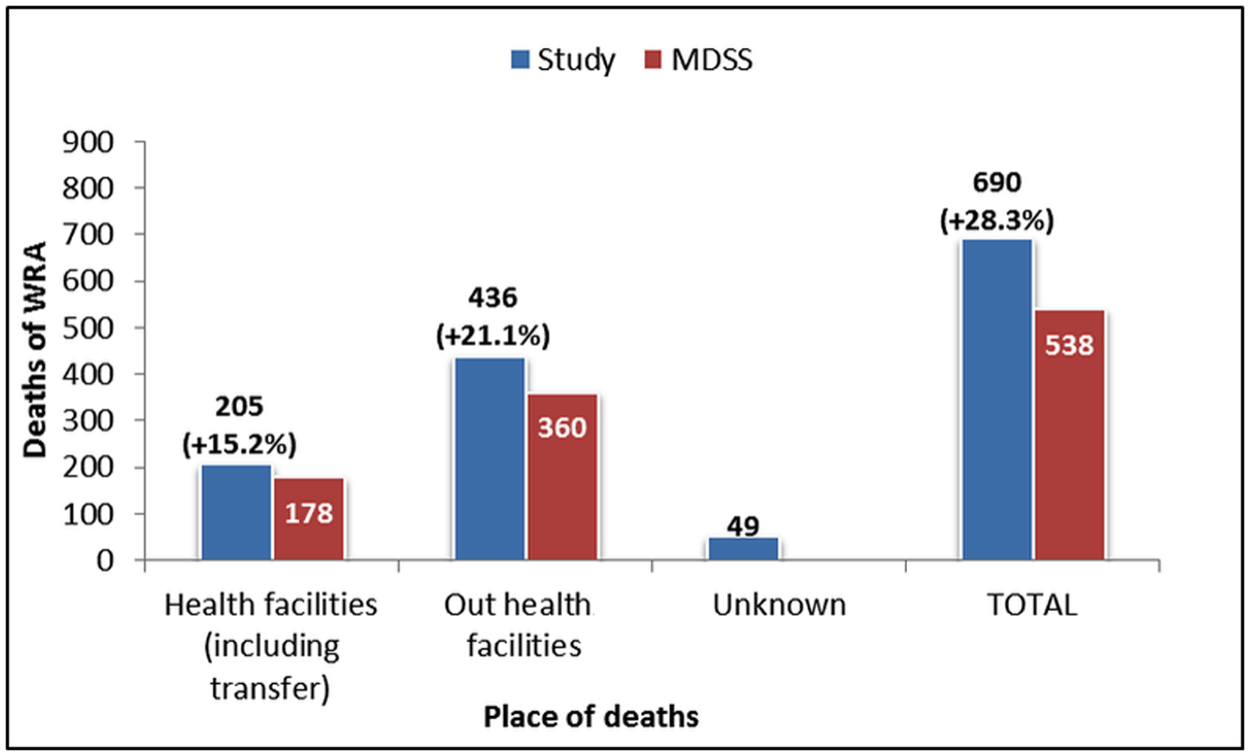
Deaths of WRA by place, identified by the study and the MDSS, 2013–2014.

Our study identified 690 deaths of WRA– 28% more than the number of deaths recorded in the MDSS. The difference in reported deaths was observed both in and outside health facilities (Fig 4).

Among the four public hospitals in the study region, the death notification system of WRA was implemented in only 3 departments (maternity ward, intensive care unit and medicine department) of one hospital. Furthermore, deaths of WRA were not systematically investigated to determine the pregnancy status of women. The hospital managers and health staff reported not being informed about the MDSS. Moreover, the focal point of MDSS at the district level (District Officer in charge of Health Programs or the person in charge of the Safe Motherhood Program) complained that the civil registration and the health offices did not report systematically, neither on time, the deaths of WRA at home.

The MDSS recorded 29 PRDs including one at home while the study identified fifty more PRDs and late maternal deaths that were not identified in the MDSS. In fact, the MDSS missed 26 (96.3%) of maternal deaths and late maternal deaths that occurred at home and 22 (39.6%) of those which occurred in health facilities. Unfortunately, it was not possible to link records between MDSS and our study database because individual questionnaires completed at the provincial level were not available. Therefore, we looked at the misclassification of PRDs and late maternal deaths in the hospital’s routine data as it was the major source of the MDSS. The study identified 51 PRDs and late maternal deaths that occurred in health facilities in addition to 4 PRDs outside health facilities but whose bodies were brought to the hospital morgue. Among those 55 deaths, 32 (58.2%) were initially identified as maternal deaths in the registry of hospital and all, except one, were due to direct causes. Twenty-three (41.8%) were classified as non PRDs (Table 4).

**Table 4:** PRDs recorded in hospitals, those identified as PRDs and those missed.

Characteristics	Study PRDs	PRDs recorded in hospital registry	Difference between study and hospital registry (%)	Number of misclassified PRDs	Percent misclassification (%)
**Place of death**					
**Public hospitals**	**45**	**26**	**57.8**	**19**	**42.2**
▪ Intensive Care Unit	24	17	70.8	7	29.2
▪ Operating theatre	7	5	71.4	2	28.6
▪ Maternity ward	5	2	40.0	3	60.0
▪ Emergencies	4	1	25.0	3	75.0
▪ General surgery department	2	0	0.0	2	100.0
▪ Department not specified	3	1	33.3	2	66.7
**Basic health service**	**1**	**1**	**100.0**	**0**	**0.0**
**Transfer between facilities**	**3**	**2**	**66.7**	**1**	**33.3**
**Private clinic**	**2**	**0**	**0.0**	**2**	**100.0**
**At home/on the way to hospital**	**4**	**3**	**75.0**	**1**	**25.0**
**Period of death**					
**During pregnancy**	**4**	**1**	**25.0**	**3**	**75.0**
**During delivery**	**2**	**1**	**50.0**	**1**	**50.0**
**Postpartum/postabortum**	**49**	**30**	**61.2**	**19**	**38.8**
▪ ≤ 24h	28	20	71.4	8	28.6
▪ > 24h and < 42d	12	7	58.3	5	41.7
▪ > 42d	5	0	0.0	5	100.0
▪ Unknown	4	3	75.0	1	25.0
**Cause of deaths**					
**Direct**	**45**	**31**	**68.9**	**14**	**31.1**
▪ Hemorrhage	24	19	79.2	5	20.8
▪ Pre-eclampsia/Eclampsia	6	4	66.7	2	33.3
▪ Hellp syndrome	4	4	100.0	0	0.0
▪ Abortion	4	2	50.0	2	50.0
▪ Infections	2	1	50.0	1	50.0
▪ Complications of care	2	1	50.0	1	50.0
▪ Direct cause undetermined	2	0	0.0	2	100.0
▪ Pulmonary embolism	1	0	0.0	1	100.0
**Indirect**	**3**	**1**	**33.3**	**2**	**66.7**
**Undetermined**	**1**	**0**	**0.0**	**1**	**100.0**
**Incidental cause**	**1**	**0**	**0.0**	**1**	**100.0**
**Late maternal deaths**	**5**	**0**	**0.0**	**5**	**100.0**
**TOTAL**	**55**	**32**	**58.2**	**23**	**41.8**

The regional MMR in this study was 103 per 100,000 live births–approximately 2.5 times higher than the MMR calculated from MDSS data for the same period (43 per 100,000 live births). The WRA mortality rate was 0.80‰ and the proportion of maternal deaths among deaths of WRA was 10.0% (S3 Table).

## Discussion

### Underreporting of deaths

#### Women of reproductive age

Only 538 (78%) WRA deaths were identified by the MDSS compared to 690 in our study. Even in health facilities, the identification of WRA deaths was lower than in our study (178 vs 205, i.e. 87%). This is a problem since the deaths of WRA are the source for the identification of maternal deaths. When focused only on certified maternal deaths MDSSs miss not only the death of pregnant women at an early stage (e.g. following abortion or ectopic pregnancy) as well as late maternal deaths, but may also lead to additional misclassification [21]. The WHO uses the proportion of maternal deaths among WRA deaths in its modeling of MMR estimates since “it is less affected by underreporting of all-cause deaths”, even excluding “studies that do not report the total number of all-cause deaths among WRA” [1].

In our study, information about the majority of deaths of WRA occurring in GCBH was obtained through civil registration offices coupled with hospitals’ and health offices’ registers. This allowed us to get a more complete picture of all-cause deaths and therefore, to improve our detection of maternal deaths.

#### Maternal deaths

In the hospitals, 32 maternal deaths were recorded in the registries and all, except one, were due to direct causes. Twenty-three deaths were classified as non PRDs even though the physicians stated that the woman was pregnant or postpartum at the time of her death. In our study, the pregnancy status was not specified in the medical records for 179 women. Several reasons have been cited to explain the underestimation of maternal deaths, including neglect by physicians, incomplete information, intention to avoid litigation or blame, and intentional suppression of information, in particular with respect to abortion deaths [1].

Misclassification of maternal deaths is a worldwide problem even in high income countries with complete and accurate vital registration systems [1, 22]. This misclassification has been estimated, and a correction factor from 0.85 to 5.0 with a median value of 1.5, is routinely added to maternal deaths identified [1, 23]. The proportion of underreporting of PRDs in our study was quite similar to that in the study conducted in Jamaica (76%) and the Philippines (75%) [24, 25]. Late maternal deaths represented 11.4% of all deaths occurring during pregnancy within one year of pregnancy, while the MDSS identified none. An active search for cause of death led to reclassification of 22.0% of maternal deaths as late in Argentina, 27.2% in Jamaica and 11.3% in Brazil [26].

Of the 360 deaths of WRA in the community, the MDSS reported only one maternal death. This calls into question the quality of the preliminary investigation by the staff at the primary health care centers.

For PRDs reported by hospitals in MDSS, a questionnaire entitled ‘confidential audit’ was completed (n = 28). However, delays in transmission of information by the hospital, limited access to medical records, which are often incomplete, and unavailability of the staff that took care of the deceased represent barriers to complete data collection.

### Areas of improvement

The notification system for maternal deaths can be improved by a combination of all sources in the region. Better communication and more advocacy efforts are essential for better collaboration between all stakeholders. The health office staff seems a strong stakeholder at the provincial level as it validates all hospital deaths certificates. It also produces death certificates in its service area for deaths that occur outside of the hospitals. Their effective involvement in the notification system for maternal deaths, especially in urban areas, can improve the completeness of MDSS. Unfortunately, these offices covered only 11 communes among the 63 in GCBH, thus accounting for approximately half the number of expected births in the region. Consequently, in rural areas the local authority should be involved in identification deaths of WRA that are not declared to civil registration offices. Currently, information about deaths is not reported by the local authority. This represents a missed opportunity for accurate registration of deaths. The ‘Moqqadem’, who is an auxiliary of the local authority in charge of reporting any significant event in his area (about 100 inhabitants), is an important key informant for improving the maternal deaths’ notification system.

Moreover, efforts should focus on public hospitals by involving the admission and reception services in charge of the hospitals’ deaths registers. Physicians and nurses in intensive care units, operating theatres, maternity wards and emergency services should be targeted initially because 50.6% of deaths during pregnancy or within one year of pregnancy occurred in these sites.

### Generalizability of results to other regions

The study region was chosen for its predominantly rural character and low proportion of institutional deliveries (59%). Its population and births only contribute to 6% of the whole country [15–18]. Moreover, disparities in access to essential obstetric care and MDSS implementation between the regions could limit the generalizability of our findings in terms of proportion of underreporting of deaths. According to the confidential inquiry conducted in Morocco in 2010, 21 PRDs were identified in GCBH, i.e. 45% of the number of maternal deaths expected during the period given the MMR (112 / 100,000 live births) [13]. At the national level, the MDSS reported 375 PRDs i.e. 54% of the expected maternal deaths in the country [13]. Six regions (including GCBH) among 14 identified less than 50% of maternal deaths. Except one region (Oriental) which identified 82% of maternal deaths the other regions did not exceed 66% [13]. This comparison shows that GCBH is a poor performer in terms of PRDs identification but also shows that all the regions miss maternal deaths. The weaknesses identified in GCBH might be similar, although to a lesser extent, to the other regions.

### Limitations of the study

The study has a number of possible limitations. First, we only compared the numbers of deaths because it was not possible to link individual records between MDSS data and our study data and therefore to further investigate the characteristics of missed or discordant cases. Second, establishment of the diagnoses was based on information given through verbal autopsies and medical records. Some of the interviews were done up to 2 years after the death while a maximum recall period of 1 year is recommended [27, 28]. Third, we possibly missed PRDs due to exclusion of women who were normally resident in the region but died elsewhere and as a result of failure to establish the pregnancy status for 30.4% of cases. However, our study has provided the most accurate figures of maternal deaths in GCBH as we targeted all WRA deaths recorded in the region.

## Conclusion

Implementation of the MDSS in Morocco is in progress but remains incomplete. The completeness of maternal deaths recording is key for an effective MDSS. This study measured the underreporting of maternal deaths at the scale of a region in Morocco and analyzed the reasons. It is important to emphasize that the majority of the maternal deaths occurred in health facilities which indicates that women use hospitals in case of severe complications. Knowing numbers however is not enough to reach the Sustainable Development Goal and attention must be given to the regional committees in charge of auditing the cases and defining actions to be implemented to prevent further maternal deaths. Such a MDSS can only work if stakeholders at all levels are fully committed.

## Supporting information

S1 Table Criteria for study region selection. (PDF)

S2 Table Deaths among WRA identified by province of residency and place of death. (PDF)

S3 Table Number of deaths of WRA and maternal deaths identified by province and Municipality or Circle of residence, 2013–2014. (PDF)

S1 Dataset (XLSX)
