# Supplementary material for: Underreporting of deaths in the maternal deaths surveillance system in one region of Morocco
Source: PLoS One. 2018 Jan 31;13(1):e0188070. doi: 10.1371/journal.pone.0188070 (PMC5791944; doi:10.1371/journal.pone.0188070)
Supplement: S1 Table — (PDF) [file pone.0188070.s001.pdf]

S1 Table. Criteria for study region selection

Region	Population (2014) [1]					Births (2014) [2]	Instutionnal deliveries (2011) [3]	Maternal Deaths recorded by MDSS (2010) [4]	Births (2010) [5]	RMM (2010)
	Total	Urban		Rural						
01. Oued Ed-Dahab-Lagouira	142 955	106 277	74%	36 678	26%	2 763	84,5	1	4 561	22
02. Laayoune-Boujdour-Sakia El Hamra	301 744	286 327	95%	15 417	5%	5 247		5	6 525	77
03. Guelmim Es Semara	501 921	346 938	69%	154 983	31%	8 942		4	10 931	37
04. Souss-Massa-Draa	3 601 917	1 736 643	48%	1 865 274	52%	75 174	59,8	57	72 255	79
05. Gharb-Chrarda-Beni-Hssen	1 904 112	906 592	48%	997 520	52%	39 559	59,0	21	40 029	52
06. Chaouia-Ouardigha	1 893 950	983 486	52%	910 464	48%	37 703	75,4	14	38 904	36
07. Marrakech-Tensift-Al-Haouz	3 576 643	1 491 243	42%	2 085 400	58%	78 563	69,5	48	70 222	68
08. Oriental	2 097 629	1 423 031	68%	674 598	32%	38 545	73,2	32	39 036	82
09. Grand Casablanca	4 270 750	4 047 066	95%	223 684	5%	68 478	90,9	32	64 413	50
10. Rabat-Salé-Zemmour-Zaer	2 676 754	2 292 120	86%	384 634	14%	44 840	90,7	17	49 293	34
11. Doukkala-Abda	2 183 090	844 730	39%	1 338 360	61%	48 787	73,8	19	42 524	45
12. Tadla-Azilal	1 607 506	632 412	39%	975 094	61%	35 926	78,3	18	31 747	57
13. Meknes-Tafilalet	2 316 865	1 442 606	62%	874 259	38%	46 681	76,6	29	43 717	66
14. Fès-Boulemane	1 808 295	1 376 157	76%	432 138	24%	34 823	74,1	16	31 139	51
15. Taza-Al Hoceima-Taounate	1 807 036	522 455	29%	1 284 581	71%	37 786	56,8	22	37 934	58
16. Tanger-Tetouan	3 157 075	1 994 356	63%	1 162 719	37%	61 138	64,2	40	56 874	70
Total	33 848 242	20 432 439	60%	13 415 803	40%	664956	72,7	375	640 104	59

1. High Commission for Planning (HCP). Recensement général de la population et de l'habitat 2014: Population légale des régions du royaume du Maroc. HCP, Rabat, Morocco. 2014.http://www.hcp.ma/downloads/RGPH-2014_t17441.html
2. Ministère de la Santé du Royaume du Maroc. Santé en chiffres 2014. Direction de la Planification et des Ressources Financières. Ministère de la Santé du Maroc, Rabat. 2015. http://www.sante.gov.ma/Publications/Etudes_enquete/Pages/default.aspx
3. Ministère de la Santé du Royaume du Maroc. Enquête Nationale sur la Population et la Santé Familiale [EPSF 2011]. Direction de la Planification et des Ressources Financières. Ministère de la Santé du Maroc, Rabat. 2012. http://www.sante.gov.ma/Publications/Etudes_enquete/Pages/default.aspx
4. CNEAC (2010). Rapport National de l'Enquête Confidentielle des Décès Maternels au Maroc. p. 1-81. Ministère de la Santé du Royaume du Maroc, February 2013.
5. Ministère de la Santé du Royaume du Maroc. Santé en chiffres 2010 Direction de la Planification et des Ressources Financières. Ministère de la Santé du Maroc, Rabat. 2011. http://www.sante.gov.ma/Publications/Etudes_enquete/Pages/default.aspx
