# Supplementary material for: Underreporting of deaths in the maternal deaths surveillance system in one region of Morocco
Source: PLoS One. 2018 Jan 31;13(1):e0188070. doi: 10.1371/journal.pone.0188070 (PMC5791944; doi:10.1371/journal.pone.0188070)
Supplement: S2 Table — (PDF) [file pone.0188070.s002.pdf]

S2 Table. Deaths among WRA identified by province of residency and place of death

Province of residency	Place of death: Health facilities (including transfer) n (%)		Outside health facilities n (%)		Unknown n (%)		TOTAL N (%)	
KENITRA	115	(29.5)	250	(63.6)	25	(6.4)	**390**	(100)
SIDI KACEM	64	(33.5)	110	(58.2)	17	(9.0)	**191**	(100)
SIDI SLIMANE	26	(23.9)	76	(70.4)	7	(6.5)	**109**	(100)
**TOTAL**	**205**	(29.7)	**436**	(63.2)	**49**	(7.1)	**690**	(100)
