# Supplementary material for: Underreporting of deaths in the maternal deaths surveillance system in one region of Morocco
Source: PLoS One. 2018 Jan 31;13(1):e0188070. doi: 10.1371/journal.pone.0188070 (PMC5791944; doi:10.1371/journal.pone.0188070)
Supplement: S3 Table — (PDF) [file pone.0188070.s003.pdf]

S3 Table. Number of deaths of WRA and PRDs identified by province and Municipality or Circle of residence, 2013-2014

Province	Municipality (Mun.) / Circle *	Population	Women aged 15-49	Deaths of WRA n (rate‰)		PRDs n (%among WRA)	
Kénitra	Kénitra (Mun.)	431 282	112 133	158	(0.81)	12	(7.6)
	Souk El Arbaa (Mun.)	69 265	18 009	27	(0.86)	5	(18.5)
	Mehdya (Mun.)	28 636	7 445	13	(1.00)	1	(7.7)
	Ben Mansour	128 780	33 483	60	(1.02)	10	(16.7)
	Kénitra-Banlieue	117 375	30 518	35	(0.66)	2	(5.7)
	Souk Tlet El Gharb	115 106	29 928	38	(0.73)	5	(13.2)
	Lalla Mimouna	93 850	24 401	30	(0.70)	4	(13.3)
	Souk Arbaa El Gharb	77 141	20 057	24	(0.68)	4	(16.7)
	Unknown address	--	--	5	--	0	--
	Total	*1 061 435*	*275 973*	*390*	*(0.81)*	*43*	*(11.0)*
Sidi Kacem	Sidi Kacem (Mun.)	75 672	19 675	43	(1.25)	7	(16.3)
	Mechraa Bel Ksiri (Mun.)	31 497	8 189	7	(0.49)	2	(28 .6)
	Jorf El Melha (Mun.)	28 681	7 457	8	(0.61)	1	(12.5)
	Had Kourt (Mun.)	7 843	2 039	3	(0.84)	0	(0.0)
	Dar Gueddari (Mun.)	6 643	1 727	2	(0.66)	0	(0.0)
	Baht	79 187	20 589	28	(0.78)	5	(17.9)
	Ouargha	78 040	20 290	32	(0.90)	2	(6.3)
	Tilal Al Gharb	73 602	19 137	19	(0.57)	3	(16.7)
	Chrarda	73 383	19 080	26	(0.78)	1	(3.8)
	Gharb-Bni Malek	67 722	17 608	20	(0.65)	3	(15.0)
	Unknown address	--	--	3	--	1	--
	Total	*522270*	*135 790*	*191*	*(0.80)*	*25*	*(13.1)*
Sidi Slimane	Sidi Slimane (Mun.)	92 989	24 177	32	(0.76)	2	(6.3)
	Sidi Yahya El Gharb (Mun.)	37 979	9 875	14	(0.81)	1	(7.1)
	Sidi Slimane	121 536	31 599	38	(0.69)	5	(13.2)
	Kceibya	67 903	17 655	14	(0.45)	3	(21.4)
	Unknown address	--	--	11	--	0	--
	Total	*320 407*	*83 306*	*109*	*(0.75)*	*11*	*(10.1)*
**Region of GCBH**		**1 904 112**	**495 069**	**690**	**(0.80)**	**79**	**(11.4)**

** A Municipality corresponds to an urban area and a Circle corresponds to a rural area.*
